# Next Generation Microbiome Research: Identification of Keystone Species in the Metabolic Regulation of Host-Gut Microbiota Interplay

**DOI:** 10.3389/fcell.2021.719072

**Published:** 2021-09-01

**Authors:** Héloïse Tudela, Sandrine P. Claus, Maya Saleh

**Affiliations:** ^1^YSOPIA Bioscience, Bordeaux, France; ^2^ImmunoConcEpT, CNRS UMR 5164, University of Bordeaux, Bordeaux, France; ^3^Department of Medicine, McGill University, Montreal, QC, Canada

**Keywords:** microbiome, dysbiosis, keystone, metagenomics, bioinformatics, inflammation, immunity, metabolism

## Abstract

The community of the diverse microorganisms residing in the gastrointestinal tract, known as the gut microbiota, is exceedingly being studied for its impact on health and disease. This community plays a major role in nutrient metabolism, maintenance of the intestinal epithelial barrier but also in local and systemic immunomodulation. A dysbiosis of the gut microbiota, characterized by an unbalanced microbial ecology, often leads to a loss of essential functions that may be associated with proinflammatory conditions. Specifically, some key microbes that are depleted in dysbiotic ecosystems, called keystone species, carry unique functions that are essential for the balance of the microbiota. In this review, we discuss current understanding of reported keystone species and their proposed functions in health. We also elaborate on current and future bioinformatics tools needed to identify missing functions in the gut carried by keystone species. We propose that the identification of such keystone species functions is a major step for the understanding of microbiome dynamics in disease and toward the development of microbiome-based therapeutics.

## Introduction

Animals are superorganisms composed of eukaryotic and prokaryotic cells in a similar proportion along an even larger number of viruses (Sender et al., 2016). The reason for this intricate mixture of organisms spanning all kingdoms of life is that every living animal is the result of a long co-evolution between all of these organisms. Hence, within every gut lies a complex community of microorganisms composed of trillions of prokaryotic and eukaryotic microbial cells, including bacteria, fungi and archaea along a multitude of viruses (Hillman et al., 2017; Moissl-Eichinger et al., 2018). As a result, we humans carry within our microbiomes an immense reservoir of genes that perform numerous functions for our own benefit, many of which are still unknown.

There is currently no consensus about the definition of a healthy gut microbiome because of high inter-individual variability, which is influenced by numerous external factors (Rinninella et al., 2019). Nevertheless, it is generally considered that a healthy gut microbiome is a rich and diverse ecosystem acting in symbiosis with its host (van de Guchte et al., 2018). Even if there is a lack of evidence to identify a robust universal set of core healthy microbial taxa, there is a remarkable stability of microbial functions that maintain symbiosis with the host (Human Microbiome Project Consortium, 2012). Conversely, we have learned over the past 20 years that some chronic disorders are consistently associated with a shift in microbial patterns, often referred to as “dysbiosis” (Hooks and O’Malley, 2017). For example, obesity has been reported to be associated with a low Bacteroidetes/Firmicutes ratio (Turnbaugh et al., 2009). Even if the Firmicutes phylum regroups a large number of potentially beneficial bacteria, this ratio has progressively been established as a hallmark of the obese dysbiotic gut microbiome (Crovesy et al., 2020). Another feature of obesity-associated dysbiosis is a reduced microbiome diversity, as illustrated by the high proportion of fecal samples from obese individuals that fall within the “low gene count” category (Le Chatelier et al., 2013). Similarly, several chronic diseases have been associated with reduced gut microbiome diversity, such as Crohn’s disease (Manichanh et al., 2006), hypertension (Li et al., 2017) and non-alcoholic steatohepatitis (NASH) (Astbury et al., 2020).

## The Keystone Species Concept as a Driver of Microbial Diversity

An important ecological concept is that every complex ecosystem is structured by a few important species dubbed “keystones.” This term was coined in 1966 by the American ecologist Paine (1966) who identified specific sea stars as important predators that regulate the biodiversity of seashores. Since, this term has been used in various ways and with different meanings. For the purpose of this review, we adopt the definition of keystone taxa proposed by Banerjee et al. (2018): *where “microbial keystone taxa are highly connected taxa that individually or in a guild exert a considerable influence on microbiome structure and functioning irrespective of their abundance across space and time. These taxa have a unique and crucial role in microbial communities, and their removal can cause a dramatic shift in microbiome structure and functioning*” (Banerjee et al., 2018). This is a crucial concept as it shapes our understanding of the regulation of complex ecosystems, how they establish, how they remain stable over long periods of time and how they adapt to environmental changes.

Translated to the gut environment, we must first appreciate that mammals harbor not just one gut ecosystem, but a variety of ecosystems, each roughly corresponding to a different section of the gastrointestinal tract from the mouth to the rectum. Each ecosystem is regulated by a set of environmental factors such as pH, bile acid concentration and peristalsis, which have long been thought of as a barrier that segregates ecosystems from one another. However, this view has been recently challenged, as it is now proposed that oral bacteria act as a reservoir of microorganisms that pass through the gut to replenish the downstream ecosystems (Schmidt et al., 2019). Within each ecosystem, microbes interact with each other through numerous mechanisms, such as secretion of quorum sensing molecules, cross-feeding and synthesis of antimicrobial compounds. Of particular interest, quorum sensing is a cell-cell interaction mechanism used by bacteria to regulate their own population. Usually in biofilms, some bacterial cells stimulate their own growth and those of their neighboring kin, through secretion of autoinducer molecules (Mukherjee and Bassler, 2019). In the gut microbiota, it has been shown that Firmicutes use this strategy to maintain their population level (Thompson et al., 2015). Interestingly, there is emerging evidence that host cells can interfere with these bacterial signals to shape the microbial community (Mukherjee and Bassler, 2019). Yet, most of our knowledge of quorum sensing is derived from the study of pathogens and there remains numerous gaps in our understanding of its use by commensal bacteria. Inter-species syntrophy or cross-feeding occurs when a species depends on the availability of nutrients (e.g., sugars, amino acids, and vitamins) that are produced by other species. For instance, this typically involves degradation of complex molecules such as carbohydrates by specialized species that release monosaccharides in the environment. The latter are then taken up by non-degrading species for their own benefit. These mechanisms have been recently thoroughly reviewed by D’Souza et al. (2018). Inter-species cross-feeding interactions within an ecosystem cause reliance on specific microbes that carry essential functions for other species. Hence many keystone species have been described based on the identification of enzymes involved in cross-feeding interactions (Centanni et al., 2018; Table 1). These are only a few examples of the diversity of possible microbial interaction routes. For a thoughtful review of the topic, we refer the reader to the review by Pacheco and Segrè (2019).

**TABLE 1:** Non-exhaustive list of prominent keystone taxa of the human gut microbiome.

**Keystone species (in alphabetical order)**	**Function**	**Method of identification**	**Example of reported disease association in humans**
*Akkermansia muciniphila*	Mucin degrader	Empirical (Belzer et al., 2017)	Intestinal inflammation, obesity and metabolic diseases (Yassour et al., 2016)
*Bacteroides thetaiotaomicron*	Degradation of complex carbohydrates (arabinogalactan); selective BSH activity (Yao et al., 2018)	Empirical (Cartmell et al., 2018)	Unclear – Controversial association with IBD (Sitkin and Pokrotnieks, 2019)
*Bifidobacterium longum*	Degradation of complex carbohydrates, particularly Human Milk Oligosaccharides; BSH activity (Tanaka et al., 2000)	Empirical (Yu et al., 2013; Gotoh et al., 2019)	Highly prevalent in healthy newborns (Favier et al., 2003)
*Bifidobacterium pseudolongum*	Degradation of complex carbohydrates	Empirical (Centanni et al., 2018)	Highly prevalent human breast milk (Lugli et al., 2020)
*Christensenella minuta*	Stimulate ecosystem diversity (Mazier et al., 2021); acetate producer (Morotomi et al., 2012); BSH activity (Déjean et al., 2021)	Co-occurrence networks (Goodrich et al., 2014; Kumpitsch et al., 2020 ahead of publication) Empirical (Ruaud et al., 2020; Mazier et al., 2021)	Obesity and metabolic diseases (Goodrich et al., 2014); Crohn’s disease (Pascal et al., 2017)
*Faecalibacterium prausnitzii*	Butyrate producer	Presence/absence (Leylabadlo et al., 2020)	Crohn’s disease (Sokol et al., 2008), Ulcerative Colitis (Varela et al., 2013)
*Methanobrevibacter smithii*	Produces methane from H_2_ and acetate	Empirical and co-occurrence networks (Goodrich et al., 2014; Kumpitsch et al., 2020 ahead of publication)	Obesity (Goodrich et al., 2014), Crohn’s disease (Pascal et al., 2017)
*Ruminococcus bromii*	Resistant starch degrader; Butyrate producer	Empirical (Ze et al., 2012)	Highly prevalent microbe in healthy individuals (Beghini et al., 2021)

Together, these mechanisms depict a high level of inter-dependencies between bacterial species within an ecosystem. These interactions lead the ecosystem to structure around clusters of microbes that co-develop into a guild of co-abundant species. This concept was well illustrated in a recent study aimed at identifying gut bacterial species involved in post-antibiotic recovery in human cohorts. In a metagenome-wide association study, Chng et al. (2020) demonstrated that a succession of primary colonizers set the stage for late dominant species, which feed on the breakdown products of the pioneer species (Gibbons, 2020). This study identified 7 bacterial species acting as primary colonizers, with a metabolic capacity to extract carbon and energy from mucin and complex dietary carbohydrates, thus acting at the bottom of the food chain. Even if in this example most of the identified primary colonizers were abundant species, low abundance bacteria (<0.1% relative abundance) should not be neglected as they may carry essential functions that support growth of other dominant species. This concept has been very well illustrated in a study of “*Candidatus Desulfosporosinus infrequens*,” a sulfate-reducing organism found in wetlands (Hausmann et al., 2019). Although it remained in a seemingly dormant state at zero-growth over more than 7 weeks, it was reported to be in fact highly metabolically active, contributing to regulate methane production and therefore to sustain a diverse ecosystem (Hausmann et al., 2019).

In view of the intricate interplay between the gut microbiome function and its host metabolism, it is now established that loosing part of these functions is associated with a number of modern non-communicable diseases. As a consequence, techniques designed to manipulate gut dwelling microbiomes are gaining increasing attention, and several microbiome-based biotherapies are currently in development (Doré et al., 2017; Valencia et al., 2017). Hence, a deep understanding of gut microbiome ecology and how it can be durably restored is crucial for effective clinical translation. In this regard, a recent study evaluated bacterial dispersal strategies of human gut-associated microbes and classified them in five categories that may provide a guide for appropriate restoration strategies: (i) “tenacious” strains that are highly persistent among human communities, (ii) “spatiopersistent” strains that tend to be associated with specific geographical locations but colonize at a later developmental stage (i.e., not in infants), (iii) “heredipersistent” strains that tend to persist within closely related individuals such as within families and have broad geographical presence, (iv) average persistent strains, and (v) non-persistent strains (Hildebrand et al., 2021). Interestingly, the authors propose that fecal microbial transplantation may be most efficient to target tenacious and spatiopersistent taxa while heredipersistent taxa may require regular reinfections, which may therefore be best targeted through chronic single strain exposure.

## Important Metabolic Pathways Under the Gut Microbiome Influence

Beyond microbiota classification, insights on the functional impact of the microbiome are emerging from metagenomic analyses, integration with omics data sets, particularly metabolomics and *in vivo* validation studies. In this section, we review recent studies highlighting the key contribution of microbial metabolites and associated pathways in controlling host physiology. Among the multitude of metabolic activities harbored by human gut microbiomes, we focus on the roles of short chain fatty acids (SCFA), tryptophan- and cholesterol-derived metabolites, and their crosstalk with host factors, e.g., histone modifying enzymes, G proteins-coupled receptors, aryl hydrocarbon receptor (AhR), indoleamine 2,3-dioxygenase 1 (IDO1) and tryptophan hydroxylase 1 (TpH1) in barrier maintenance, immune regulation, and the gut-brain axis.

### SCFAs

Short chain fatty acids are the primary end products of bacterial fermentation of dietary fibers (but can also be derived from proteins and peptides in a lesser extent) and are important regulators of gut microbial ecology as well as host physiology. The main SCFAs are acetate, propionate, and butyrate (Cummings et al., 1987). Fiber-derived monosaccharides, such as hexoses, deoxyhexoses, and pentoses are converted by several bacterial metabolic enzymes to pyruvate which is then further metabolized to acetyl-CoA, succinate or lactate that primarily feed SCFA production. Acetate is derived from acetyl-CoA generated from pyruvate directly or through the Wood-Ljungdahl pathway. Butyrate is also produced from acetyl-CoA, but through the condensation of two acetyl-CoA molecules into acetoacetyl-CoA that is metabolized to butyryl-CoA and then butyrate. Some gut bacteria can also convert lactate to butyrate. Propionate is derived from lactate or succinate in the acrylate and succinate pathways, respectively. It can also be produced by the propanediol pathway that converts deoxyhexoses to proprionyl-CoA. The concentration of SCFAs is highest in the proximal colon reaching ∼130 mmol/kg of luminal content (Cummings et al., 1987). However, the effective concentration reaching the intestinal cells is presumably lower due to the thick mucus layer and intestinal peristalsis. Among the SCFAs, butyrate constitutes an important energy source for colonocytes and is mostly consumed in the colon. Propionate and acetate are further metabolized in the liver, but taken the high concentration of acetate in the gut, it is the main SCFA that remains in the systemic circulation. Nonetheless, butyrate and propionate can also impact host systemic physiology indirectly through hormonal and nervous system signals. SCFAs can enter the cells though diffusion or via the transporter SLC5A8 and exert their effects through three reported mechanisms: (a) epigenetic control of gene expression via inhibition of histone deacetylases (HDAC), e.g., in intestinal epithelial cells (IECs) and immune cells, (b) by acting as ligands of G-protein coupled receptors (GPCRs), primarily GPR43 and GPR41, also called free fatty acid receptors 2 (FFAR2) and FFAR3, respectively, and GPR109A, also known as niacin receptor 1 or Hydroxycarboxylic Acid Receptor 2 (HCA2), and/or (c) by acting as an AhR agonist, as has been shown for butyrate in IECs (Marinelli et al., 2019).

**FIGURE 1:**
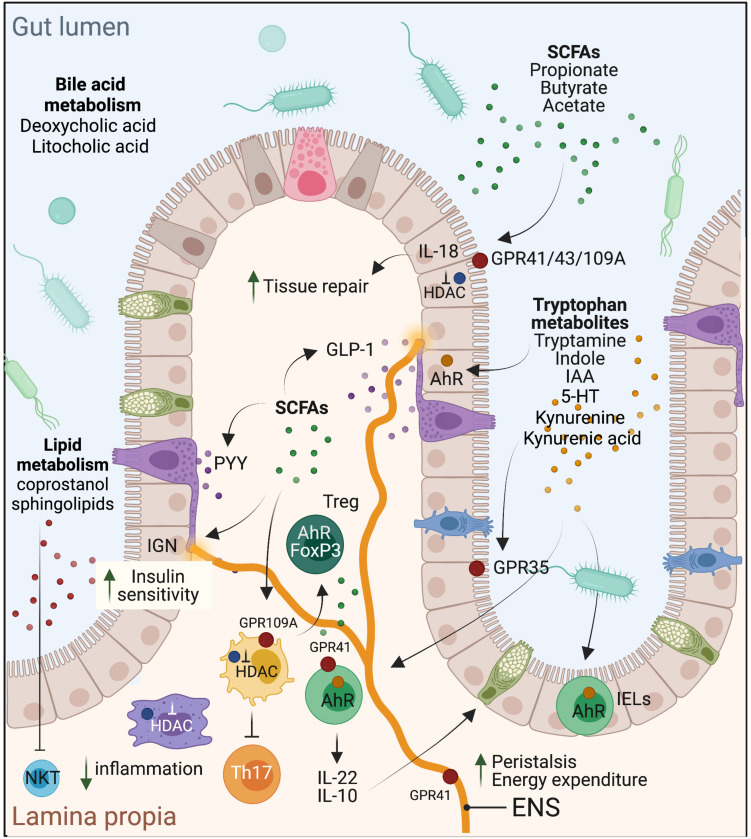
Schematic representation of the effects of select microbial metabolites on intestinal homeostasis, mucosal immune regulation, and metabolic health of the host.

Because of the energetic reliance of colonocytes on butyrate, it is not surprising that this SCFA is a critical regulator of intestinal barrier integrity and mucosal immune homeostasis. Butyrate confers a protective role in experimental mouse models of colitis [e.g., with dextran sodium sulfate (DSS)], *Il10^–/–^* mice (Wang et al., 2016) or *Clostridium difficile* infection (Fachi et al., 2019). These effects were also noted in ulcerative colitis patients, as shown early on by Scheppach et al. (1992). Butyrate also protects against colitis-associated colorectal cancer (CRC) as has been reported using the *APC^*min/*+^* mice (Singh et al., 2014) or with the azoxymethane (AOM)-DSS model (Singh et al., 2014). In contrast, in the *APC^*min/*+^MSH2^–/–^* mouse model with stem-like CRC characteristics, butyrate was shown to promote tumorigenesis (Belcheva et al., 2014), presumably through enhancing stem cell regeneration. Mechanistically, butyrate signals through GPR43 and GPR109A on IECs to stimulate inflammasome-dependent IL-18 production (Macia et al., 2015), which is required for intestinal epithelial integrity (Dupaul-Chicoine et al., 2010; Figure 1). It also protects from colonic inflammation through HDAC inhibition that blunts lamina propria macrophages inflammatory signaling (Chang et al., 2014) and dendritic cells differentiation (Millard et al., 2002; Wang et al., 2008; Singh et al., 2010), and promotes regulatory T cells (T_reg_) generation (Arpaia et al., 2013; Furusawa et al., 2013), through acetylation of the *FoxP3* locus (Figure 1). More recently, butyrate, in addition to propionate and acetate, was shown to induce IL-22 expression in CD4^+^ T cells and innate lymphoid cells (ILC) through GPR41 and HDAC inhibition; the latter enabling enhanced binding of HIF1α to the *Il22* gene promoter (Yang et al., 2020). In cancer cells, which favor glucose metabolism (Warburg effect), butyrate was shown to accumulate in the nuclei leading to effective inhibitory concentrations of HDACs (Donohoe et al., 2012). As a result, butyrate can epigenetically deregulate the expression of key genes involved in cell proliferation, cell death and differentiation in cancer cells but not normal colonocytes (Donohoe et al., 2012). Propionate, but not acetate, similarly promotes these processes through HDAC inhibition. A metagenomic-based approach was able to identify the main butyrate producers of the gut microbiome as *Eubacterium rectale*, *Faecalibacterium prausnitzii*, and *Anaerostipes coli* S22/1 (Louis et al., 2010; Muñoz-Tamayo et al., 2011). Yet, only *F. prausnitzii* was formally identified as a keystone species (Table 1). Interestingly, *E. rectale* was identified in another study using metagenomic time series as a bacterium benefiting from the presence of putative keystone species such as *Bacteroides fragilis and Bacteroides stercosis* (Fisher and Mehta, 2014).

In metabolism, dietary fibers and SCFAs are generally associated with lean weight and improved glycemic index. For example, an improvement in insulin sensitivity was reported in a trial in which individuals were given a diet supplemented with a resistant starch for 4 weeks (Robertson et al., 2005). This beneficial effect can be attributed to SCFA. In a randomized controlled trial, administration of an inulin-propionate ester to overweight adult humans over 24 weeks reduced body weight gain, abdominal adiposity and hepatic lipid accumulation compared to the inulin-control group (Chambers et al., 2015). Similarly, colonic infusion of SCFA mixtures in overweight/obese men, at concentrations comparable to those reached after dietary fibers intake, increased fat oxidation and energy expenditure (Canfora et al., 2017). Mechanistically, SCFAs may act by stimulating the production of the anorexigenic gut hormones peptide YY (PYY) and glucagon-like peptide-1 (GLP-1) (Figure 1), as has been shown in humans with acetate (Freeland and Wolever, 2010) and propionate (Venter et al., 1990) or through intestinal gluconeogenesis (IGN), where both propionate and butyrate were shown to enhance IEC *de novo* synthesis of glucose, stimulating increased insulin sensitivity through gut-brain communication (De Vadder et al., 2015). GPR41 mediates improved energy metabolism through its expression on neurons of the enteric nervous system (ENS) and on sympathetic neurons that promote enhanced energy expenditure (Figure 1). Consistently, *Gpr41^–/–^* mice were shown to be leaner than wild-type controls (Samuel et al., 2008). On the other hand, the results with *Gpr43^–/–^* mice are controversial as these mice were described to be obese even on normal diet in one study (Kimura et al., 2013), but lean with improved metabolic parameters in another (Bjursell et al., 2011). Nonetheless, these mouse phenotypes were lost under germ-free conditions or with antibiotic treatment, demonstrating microbiota-mediated metabolic effects.

### Tryptophan Metabolism

Microbial metabolism of dietary tryptophan (enriched in cruciferous green leaf vegetables, e.g., parsley, cauliflower, kale, broccoli, etc.) has recently emerged as an important pathway by which the microbiota regulates intestinal homeostasis, particularly through AhR activation. Since its cloning in 1992 (Burbach et al., 1992), AhR has gained much interest for its role as an environmental sensor. Beyond its activation by xenobiotics, tryptophan-derived ligands catabolized by the microbiota, including indole, indolo[3,2-b]carbazole, indole acetic acid (IAA), 3-methylindole and tryptamine, to name a few, have been demonstrated to act as potent high-affinity AhR ligands (Zelante et al., 2013; Hubbard et al., 2015). AhR is expressed in different intestinal cell types, including IECs, immune cells, particularly intraepithelial lymphocytes (IELs), innate lymphoid cells (ILC)3 (Gomez de Agüero et al., 2016), more recently ILC2 (Li et al., 2018), Th17 and T_reg_ (Quintana et al., 2008; Veldhoen et al., 2008), and neurons of the ENS (Obata et al., 2020). Through this collective expression, AhR exerts physiologically crucial roles in barrier integrity and intestinal and immune homeostasis, notably through regulation of IEC tight junctions (Yu et al., 2018; Singh et al., 2019), generation and survival of IELs (Cervantes-Barragan et al., 2017), production of IL-22 (Zelante et al., 2013) and IL-10 (Aoki et al., 2018; Powell et al., 2020), and regulation of peristalsis and microbiota density (Figure 1). In IECs, AhR has been recently implicated in the regulation of goblet cells differentiation, particularly in preventing goblet cell depletion in the colon during aging (Powell et al., 2020). This process is mediated by IL-10 and induced by AhR in response to microbiota-derived indoles. IL-22 or type I IFN, which are involved in intestinal tissue repair following acute injury, do not seem to be required in this case (Powell et al., 2020). In parallel to IECs, AhR activation in CD4 + T cells was shown to regulate their differentiation into CD4 + CD8αα + double-positive immunoregulatory IELs. These cells are absent in germ-free mice but restored with *Lactobacillus reuteri*, a species with tryptophan catabolizing capacity (Cervantes-Barragan et al., 2017). AhR activity is similarly required for the generation and maintenance of ILC3 (Gomez de Agüero et al., 2016), and patients with Crohn’s disease exhibit decreased AhR expression in their inflamed ileum accompanied by a conversion of ILC3 to ILC1 (Li et al., 2016). Last, AhR expression is elevated in intestinal T_reg_ and seems to be required for their gut homing as well as for suppression of Th1 inflammatory gene expression (Ye et al., 2017). AhR is also expressed in colonic neurons, in a microbiota-dependent manner, and this neuronal-specific expression is central in the control of intestinal peristalsis, positioning AhR as a nexus of intestinal neural circuitry (Obata et al., 2020). Collectively, AhR protects the epithelial barrier, promotes intestinal immune tolerance and protects from intestinal inflammation. Consequently, deregulation of its activity is associated with inflammatory and metabolic diseases, and microbiome stimulation of this pathway, particularly through tryptophan metabolism, has been demonstrated as an “environmental” mean to counter these pathologies. For instance, individuals with inflammatory bowel diseases (IBD) (Lamas et al., 2016), the metabolic syndrome (Natividad et al., 2018) or celiac disease (Lamas et al., 2020) have decreased fecal concentrations of AhR ligands and reduced AhR activity. Interestingly, supplementation of experimental mice modeling these pathologies with a high-tryptophan diet, AhR ligands or with bacterial species that metabolize tryptophan such as *L. reuteri*, improved the mice health status (Marafini et al., 2019; Chen et al., 2020). In a randomized controlled clinical trial, administration of AhR ligands in the form of the traditional Chinese medicine indigo naturalis to ulcerative colitis patients for 8 weeks showed clinical benefit, including a decrease in the Mayo score, mucosal healing and remission in some cases (Naganuma et al., 2018). Nonetheless, caution is warranted prior to considering the development of AhR ligands for therapeutics taken toxicity issues with deregulated AhR responses.

Besides AhR ligands, tryptophan is additionally metabolized into kynurenine and serotonin (Clarke et al., 2012; Yano et al., 2015). In the kynurenine pathway (KP), IDO1 is mainly responsible to convert tryptophan to kynurenine and downstream end products including niacin, nicotinamide adenine dinucleotide (NAD), quinolinic acid and kynurenic acid (Cervenka et al., 2017; Kennedy et al., 2017). The latter exerts immunoregulatory effects and protects the intestine by signaling through GPR35, expressed on IECs and immune cells (Gao et al., 2018). The serotonin pathway converts tryptophan into the neurotransmitter 5-hydroxytryptamine (5-HT), i.e., serotonin, via TpH1 expressed in a specialized IEC type known as enterochromaffin cells in the gut, and TpH2 in the brain. While peripheral 5-HT, which constitutes 90% of all serotonin produced in the body, does not cross the blood–brain barrier (BBB), it regulates several intestinal processes including stimulation of ENS neurons, peristalsis and nutrient absorption, to name a few (Mawe and Hoffman, 2013). Moreover, both tryptophan and 5-HT precursor (5-HTP) cross the BBB impacting central serotonin effects on host physiology. The commensal microbiota plays important roles in tryptophan metabolism to kynurenine and serotonin as demonstrated in GF or antibiotics-treated mice [reviewed in Agus et al. (2018)]. Several commensal bacteria express enzymes related to KP enzymes and can thus produce kynurenine metabolites, e.g., the neurotoxic 3-hydroxyanthranilic acid (O’Farrell and Harkin, 2017). Further, through SCFA and BA metabolism, the microbiota can induce TpH1 and stimulate 5-HT biosynthesis (Reigstad et al., 2015; Yano et al., 2015). Gut-derived kynurenines and 5-HT are additionally implicated in the pathogenesis of chronic inflammatory, metabolic and neuropsychiatric diseases. For instance, IDO1^–/–^ mice are more susceptible to 2,4,6-trinitrobenzene sulfate-induced colitis (Takamatsu et al., 2013) whereas TpH1^–/–^ mice show enhanced protection in response to DSS- or dinitrobenzene sulfonic acid-induced colitis (Ghia et al., 2009), suggesting that while kynurenine is protective in the gut, 5-HT might be deleterious. However, more recent findings indicate that 5-HT could exert pro- or anti-inflammatory effects in the gut depending on the respective engagement of 5-HT7 versus 5-HT4 receptors (Spohn and Mawe, 2017). Kynurenines and 5-HT play contrasting roles in obesity and insulin resistance. The KP is overactivated in obesity and its activity correlates with BMI and the metabolic syndrome, presumably through the action of kynurenine derivatives such as xanthurenic acid (Oxenkrug, 2013). In contrast, 5-HT levels are decreased in obese individuals, which is consistent with the role of 5-HT in promoting satiety (Voigt and Fink, 2015), lipolysis in white adipose tissue and hepatic gluconeogenesis (Sumara et al., 2012).

### Cholesterol and Lipid Metabolism

Cholesterol and lipid metabolism by the intestinal microbiota is an additional facet by which the microbiota influences host physiology. On one hand, microbial components, specifically *Peptostreptococcus anaerobius*, have been identified as inducers of cholesterol biosynthesis in colonocytes, mediated by SREBP2 activation downstream of TLR signaling, which supports dysplasia and CRC development in a mouse model, and is consistent with elevated levels of this bacteria in the stool of CRC patients (Tsoi et al., 2017). On the other hand, microbial metabolism of cholesterol into coprostanol, a poorly absorbed sterol, has been recently demonstrated as a mechanism reducing host serum cholesterol levels (Kenny et al., 2020). Notably, this function was attributed to a clade of uncultured bacteria harboring ismA genes encoding cholesterol dehydrogenases (Kenny et al., 2020). Besides cholesterol, bacterial metabolism of sphingolipids (SL), lipids with a long-chain amino alcohol backbone, also contributes to immune homeostasis in the gut and to the host metabolic health. Bacteria of the Bacteroidetes phylum, which constitutes ∼30–40% of the healthy human intestinal microbiota, have the capacity to synthesize sphingolipids (SL), owing to their expression of the enzyme serine palmitoyltransferase (Heaver et al., 2018). Bacterially derived SL promote immune homeostasis in the gut, acting early in life to tame invariant natural killer T (iNKT) cells proliferation (An et al., 2014). Consistently, in a model of oxazolone-induced colitis, treatment of mice with *B. fragilis* SL lessened the colitis phenotype by reducing iNKT cell numbers (An et al., 2014). Notably, the stools of IBD patients contain an elevated signature of host SL including ceramides, but are depleted of bacterially derived SL which protect the intestine. Indeed, colonization of germ-free mice with *Bacteroides thetaiotaomicron* deficient in SL elicited intestinal inflammation (Brown et al., 2019). To address how the gut microbiota influence host metabolic pathways and ceramide levels, a recent study explored this question in a model of diet-induced obesity (Johnson et al., 2020). The authors showed that bacterially derived SL can enter colonocytes and reach the liver through the portal vein circulation, impacting metabolic parameters, e.g., insulin resistance, primarily through liver ceramides (Johnson et al., 2020). Together, these indicate that cholesterol and lipid metabolism by gut bacteria significantly influence host metabolism and physiology. However, these pathways are still poorly understood and require to be fully investigated before further therapeutic exploitation.

### Bile Acid Metabolism

Bile acid metabolism by the gut microbiota has gained considerable interest in the recent years as they are being recognized as crucial microbiome-derived metabolites that regulate multiple important functions involved in health and disease (Hylemon et al., 2018). Primary bile acids are mostly synthesized by the liver hepatocytes from cholesterol following irreversible 7alpha-hydroxylation by the cytochrome P450 CYP7A1 (Chávez-Talavera et al., 2017). In humans, these are cholic acid (CA) and chenodeoxycholic acid (CDCA), while in mice, CDCA is further metabolized into beta-muricholic acid (betaMCA) (Pandak and Kakiyama, 2019). These hydrophobic primary bile acids are then made amphipathic through conjugation with glycine and taurine before being secreted in the gall bladder along phospholipids to make up the bile that will primarily serve as detergent upon release into the duodenum during digestion (Chávez-Talavera et al., 2017). In the small intestine, conjugated bile acids are deconjugated by microbial bile salt hydrolases (BSH) that release the hydrophobic moieties that are reabsorbed mostly through passive diffusion along the epithelium and through active reabsorption in the terminal ileum. In total, 95% of the initial bile acid pool is reabsorbed through this enterohepatic cycle. Yet, 5% of bile acids escape reabsorption and travel down the colon where they undergo further metabolism by gut bacteria such as 7alpha-dehydroxylation, which leads to formation of secondary bile acids such as deoxycholic acid and litocholic acid from the metabolism of CA and CDCA, respectively, (Ridlon et al., 2016). This is a brief overview of the complex metabolism of bile acids. For an exhaustive review of the role of gut microbes on bile acid metabolism, we refer the reader to the work by Ridlon et al. (2016). Gut microbial taxa with documented BSH activity include *Lactobacillus* spp. (Foley et al., 2021), *Bifidobacterium longum* (Tanaka et al., 2000), *Enterococcus faecalis* (Chand et al., 2018), *B. thetaiotaomicron* (Yao et al., 2018), and *Christensenella minuta* (Déjean et al., 2021), some of which have been classified as keystone species (Table 1).

Beyond their role in lipid absorption, bile acids have systemic functions as they also regulate hepatic energy metabolism, adipocyte differentiation and dampen immune activation through their interaction with bile acid receptors Farnesoid X Receptor (FXR) and Takeda G protein Receptor 5 (TGR-5) (Foley et al., 2021). Because of these multiple effects, they were recently suggested to form gut microbiota-derived hormones (Kliewer and Mangelsdorf, 2015; Hylemon et al., 2018). Both conjugated and unconjugated bile acids participate in this host-microbiota crosstalk. Interestingly, new bile acid conjugates specifically produced in the small intestine, were recently discovered. These involve bacterial conjugation with phenylalanine, tyrosine, and leucine, three hydrophobic amino acids, which had never been described associated with these molecules. Unsurprisingly, we still ignore the physiological role of these novel microbially derived compounds (Quinn et al., 2020).

Bile acids have been associated with a number of chronic disorders including obesity, NASH, IBD (Schirmer et al., 2019), Primary Biliary Cholangitis (formerly known as Primary Biliary Cirrhosis) and Primary Sclerosing Cholangitis. Interestingly, some disorders have been specifically associated with a defect in gut bacterial metabolism of bile acids such as *Clostridioides difficile* infections, which have been shown to be corrected by restoring gut microbial BSH activity (Mullish et al., 2019). Indeed, bile acid deconjugation releases free unconjugated bile salts that are toxic to most bacteria and thus act as a regulator of the microbial ecosystem. Hence, this is one of the key functions carried by gut bacteria that impacts significantly on the composition of the gut microbiome community.

## Bioinformatic Tools to Identify Keystone Species and Functions of the Gut Microbiome

In light of these findings, restoring key metabolic pathways carried by gut microbiota could open a wide range of therapeutic perspectives. In particular, the identification of keystone species carrying these functions in the gut microbiome appears as an essential step for the development of future biotherapies targeting the gut microbial ecosystem. Keystone species of the human microbiome have often been identified using empirical evidence (Banerjee et al., 2018). Nevertheless, bioinformatics is increasingly used to identify keystone species and several methods have been developed to exploit next-generation sequencing (NGS) data.

### “Presence or Absence” Associated With Health and Disease

One of the most common methods to characterize the human gut microbiome has been the use of amplification and sequencing of marker genes in stool samples. The most used marker gene is the 16S rRNA gene for the detection of bacteria, but other housekeeping genes are occasionally used to capture bacterial diversity (Case et al., 2007; Ogier et al., 2019). 16S rRNA can be used to compare the abundance of microorganisms at the genus or species level in different states (e.g., healthy versus diseased, different food sources, etc.) by assigning the reads to clusters of organisms grouped by taxonomic marker gene similarity, called Operational Taxonomic Units (OTUs). A widely used technique to then assess variations between microbial communities is to use UniFrac beta-diversity metric coupled with unsupervised multivariate statistics using Principal Component Analysis (PCA) or its derivatives (Lozupone and Knight, 2005; Ramette, 2007). However, the main hurdle associated with the calculation of the UniFrac method is the high computer power required, although this has been largely improved in the latest Striped UniFrac algorithm (McDonald et al., 2018). More complex probabilistic methods, such as Dirichlet multinomial mixtures, have been developed to improve the analysis of metagenomics samples by clustering and classifying microbial communities based on a probability distribution. This method especially considers the discrete nature, the sparsity and the variability of the sequencing depth, and has been applied to the analysis of samples from obese and lean twins and to IBD patients (Holmes et al., 2012; Ding and Schloss, 2014).

Even if evaluating the presence or absence of a genus or species between two states using marker gene sequencing in stool samples seems promising, a key part of the keystone species definition is the interspecies interaction (Banerjee et al., 2018). Hierarchical clustering of bacterial communities correlated with disease association have been extensively used to attempt identification of important bacterial taxa (Jackson et al., 2018). Correlation with taxon presence or absence is often confirmed using metrics such as Jaccard’s index: between two species, this index is defined by the ratio between the number of samples where both species are present out of the number of samples tested. The values of this index range between 0 and 1, from no correlation to a strong correlation, respectively, (Mainali et al., 2017). Nevertheless, an extensive study on microbial community modeling showed that indexes selected to evaluate correlations between species should be adapted to the specific dataset being studied. It is noteworthy that Jaccard index has been reported to have a relatively low sensitivity compared to other metrics such as the Pearson index when applied to co-occurrence networks (Berry and Widder, 2014). Therefore, even if the use of Jaccard’s index is reliable to identify the correlations using taxon presence or absence, the use of Pearson or Spearman indexes should be preferred to assess correlations when using network-based methods.

To overcome the issues associated with taxon-based correlation analysis that may lead to conflicting results due to spurious associations, it is noteworthy that Wu and co-authors have recently proposed to exploit the concept of bacterial guild to reduce metagenomic data dimensionality into ecologically meaningful functional units (Wu et al., 2021). Although this approach is still limited by the use of relative abundance data, it proposes an interesting approach to refine data analysis of 16S-based datasets to identify relevant disease associations.

### Prediction of Interspecies Interactions by Network-Based Methods

The need to consider interspecies interactions to identify keystone species in a community led to the development of new network-based methods. The most common methods are co-occurrence or co-abundance networks applied to 16S rRNA gene (or other marker gene) sequencing or metagenomic data. These networks are often produced by calculating a pairwise correlation coefficient between each pair of OTUs but other methods are being developed to build interaction networks (Berry and Widder, 2014).

Co-occurrence networks have been extensively used to identify keystone species. An exhaustive study by Berry and Widder (2014). evaluated the performance of these networks in interaction with several correlation metrics. They used generalized Lotka-Volterra modeling (gLVM) to simulate competitive and cooperative interactions between species (Berry and Widder, 2014). This study revealed that high mean degree (the average number of edges a node has in the network), high closeness centrality (the average distance of a node to any other node), high transitivity and low betweenness centrality (the betweenness centrality of the node A is the number of shortest paths between a pair of nodes B and C passing through the node A) can predict the keystone nature of species with 85% accuracy (Berry and Widder, 2014). These parameters produce highly interconnected nodes (i.e., keystone species) called “hub” patterns corresponding to a number of species interacting together directly and indirectly (Berry and Widder, 2014; Layeghifard et al., 2017; Banerjee et al., 2018). This enables identification of the guilds of bacteria that depend on the presence of keystone species. For example, this method was used by Zhang et al. (2014) on 454-pyrosequencing 16S rRNA sequencing data from human intestinal biopsy samples. It allowed to identify *Ruminococcus gnavus*, *Faecalibacterium prausnitzii*, *Prevotella copri*, and *Anoxybacillus flavithermus* as potential keystones of a healthy human intestinal mucosal microbiota because they displayed the highest number of linkers (Zhang et al., 2014). As already mentioned, only *F. prausnitzii* has been so far empirically validated as a keystone species for its ability to produce butyrate (Table 1). Its loss has been associated with the development of IBD in several studies (Sokol et al., 2008; Varela et al., 2013).

Another study from Fisher and Mehta (2014) used discrete-time Lotka-Volterra models to simulate the abundance variations of 10 species for 1000 timesteps and 100 initial conditions. After demonstrating that correlations in species abundance were not predictive of interactions between species, they used this simulated dataset to prove that LIMITS, an algorithm that uses sparse linear regression corrected by bootstrap aggregation, can be relevant to identify keystone species in two time-series samples from the gut microbiome (Fisher and Mehta, 2014). They concluded that *B. stercosis* and *B. fragilis* showed more interactions than other bacteria and that these interactions were mainly beneficial, since the growth of these two bacteria has been correlated with an increased abundance of *B. thetaiotaomicron* and *E. rectale*, the latter being a well-known butyrate producer (Fisher and Mehta, 2014). These results are coherent with other studies identifying *Bacteroides sp.* as key members of the gut microbiome (Loftus et al., 2021) involved in the degradation of complex carbohydrates (Cartmell et al., 2018). Nevertheless, the major drawback of this method is the need of time-series samples which are difficult and time-consuming to obtain. Furthermore, only a few abundant species are studied, thus the identification of low abundant keystone species is fairly unlikely using this method.

Other network-based methods, such as association rule-mining, are being applied to microbiome sequencing data in order to identify keystone species. Briefly, this method allows to estimate whether the presence of a species is required for the presence of other species. In a study by Chng et al. (2020), using the “efficient_apriori” Python package, they inferred binary association rules between species on 782 microbiome profiles. This method allowed to identify “primary” species, which presence is required for other species to thrive. Furthermore, they showed that some of these “primary” species, such as *Bacillus uniformis*, *F. prausnitzii* or *Ruminococcus torques*, were associated with the recovery of the gut microbiota after antibiotic exposure and carried specific metabolic functions such as mucin and carbohydrates degradation (Chng et al., 2020). As mentioned in Table 1, other members of the *Ruminococcus* genus have been identified as resistant-starch degraders (Ze et al., 2012) essential to maintain a healthy gut microbiome (Beghini et al., 2021).

Co-occurrence networks between members of the microbiota can also be applied using generalized boosted linear models (GBLMs), as exemplified in a study where it was implemented to investigate the Human Microbiome Project cohort (Faust et al., 2012). More recently, a co-occurrence network using the Random Matrix Theory (RMT) method was applied to murine gut microbiome data in order to identify putative keystone species. In this study, 32 were identified as highly connected species linked to other OTUs (Liu et al., 2019).

Although these methods are useful to predict putative keystone species, the identification of the keystone functions carried by these species is essential to understand the interactions between the keystone bacteria and its guild. However, the use of amplicon sequencing does not allow the precise identification of the strains or the metabolic functions they carry within the gut microbiome because the assignation of OTUs only allows the reconstruction of metabolic pathways based on reference genomes, thus inducing a possible loss of strain-specific metabolic functions (Frioux et al., 2020). Therefore, a few recent methods have been developed to overcome these issues using metagenomic sequencing that reconstruct strain-specific metabolic pathways.

### Reconstruction of Metabolic Pathways at Ecosystem Level

The recent development of metagenomics has provided a clearer view of the diversity of the gut microbiota, especially by allowing access to yet-uncultured bacteria (Almeida et al., 2019). The reconstruction of genome-scale metabolic networks and models (GSMNs) using Metagenome-Assembled Genomes (MAGs) or the inference of functional categories to single genes allows to precisely predict the metabolic capabilities of the gut microbiome (Frioux et al., 2020). A good example of such tools is the Metage2Metabo algorithm developed by Belcour et al. (2019) that enables the analysis of metabolic pathways at the ecosystem level using both reference genomes and MAGs. As keystone species carry essential metabolic functions in the gut ecosystem, this tool was used to detect putative keystone bacteria. In order to accurately predict the metabolic capabilities of the communities, both available nutrients and genome information (from reference databases or metagenomic samples) are combined to predict minimal communities of bacteria that can produce target metabolic compounds. The bacteria encountered in all predicted minimal communities are considered essential symbionts, whereas bacteria encountered in at least one of the predicted minimal communities, but not necessarily all of them, are considered alternative symbionts (Belcour et al., 2019). This method has been applied to a set of over 1,500 reference genomes from the human gut, allowing the identification of 11 essential symbionts, namely *Propionibacterium* sp. KPL2009, *Paenibacillus polymyxa*, *Bacillus licheniformis*, *Lactococcus lactis*, *Enterococcus casseliflavus*, *E. faecalis*, *Hungatella hathewayi*, *Dorea longicatena*, *R. torques*, *Burkholderiales bacterium*, and *Citrobacter portucalensis*, and 194 alternative symbionts (Belcour et al., 2019). As the reference genomes were assembled from 155 fecal samples, bacterial communities from each individual were mixed, maybe explaining the large amount of alternative symbionts (Zou et al., 2019). In addition, the fairly reduced number of predicted keystone species compared to the input dataset of genomes could be due to the non-exhaustive list of initial nutrients and target metabolic compounds that need to be provided to the software in order to predict minimal communities. Nonetheless, this tool is the first tool to the best of our knowledge that is specifically designed to identify both putative keystone species and their associated metabolic functions in complex microbial communities.

## Final Considerations

Modern sequencing technologies enable broad mapping of virtually any microbial ecosystem. Nevertheless, it is important to keep in mind that the quality of microbiome data and the information we derive from them highly rely on the quality of the original sampling. Indeed, environmental factors strongly influence bacterial communities that adapt to any variation, being the time of day, temperature, pH, food supply (i.e., diet for gut microbiomes), and the age of their host for host-associated microbiomes. In this regard, the keystone species *C. minuta* has been reported to be increased with aging (Waters and Ley, 2019). Hence, repeated sampling can be recommended in order to account for temporal variations and obtain more accurate pictures of bacterial ecosystem compositions (Ji et al., 2019).

Another important consideration about sampling is that most large human studies focus on easily accessible locations with non-invasive tools, analyzing oral and fecal samples for instance. As a consequence, the analyzed microbiomes poorly represent the inner ecosystems located deep within the gut. Typically, fecal samples reflect the microbiome of the distal colon, largely dominated by Clostridiales, while it has been shown that the gut microbiota follows a specific spatial distribution along the gastrointestinal tract, which is even further complicated by regional specificities as illustrated by the differences observed between luminal and mucosa-associated species (Donaldson et al., 2016). Hence, most studies of human cohorts are limited to the study of the distal gastrointestinal tract. Despite this limitation, NGS methods and bioinformatics have been effectively supporting the search for keystone species in the gut microbiome. The rapid improvement of NGS techniques that generates increasingly large datasets allowing for deep characterization of the gut microbiome community also calls for new bioinformatics tools to analyze NGS datasets in a more effective and complete way.

**FIGURE 2:**
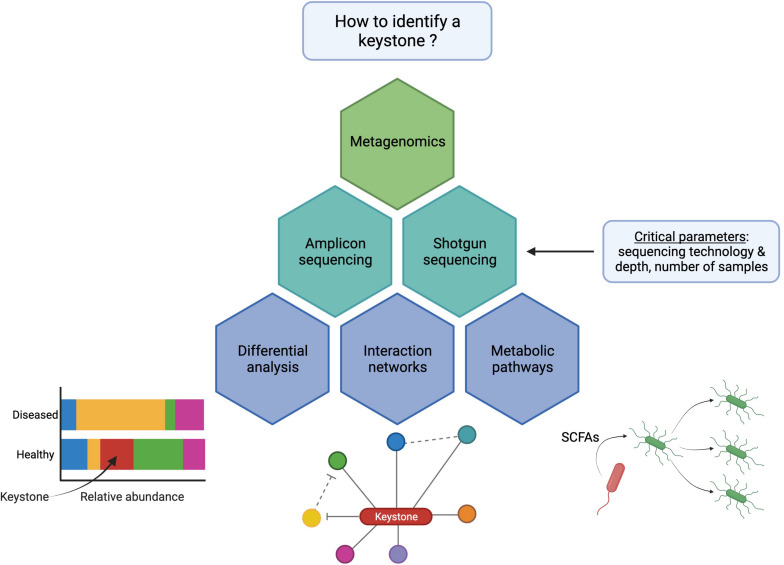
Diagram of the most used bioinformatic strategies to identify microbiota keystone species using next-generation sequencing. The bacteria shown in red is a keystone species that disappears from the stool of patients in differential analyses (bottom left), is highly connected to other species through beneficial interactions (“hub”) (bottom middle), or produces essential metabolites (e.g., SCFAs) that enhance the growth of other bacteria (bottom right).

For metagenomics studies and as summarized in Figure 2, two methodology decisions drastically influence the results: (i) the DNA sequencing technology and (ii) the bioinformatic methods that will be applied to analyze these datasets.

The choice of a DNA sequencing strategy determines the information retrieved from a sample. Amplicon sequencing, especially 16S rRNA gene sequencing, allows to have an overview of the bacterial content of the samples by assigning the obtained reads to taxonomies. Metabolic networks can then be inferred using the reference genomes of the species or genus identified in the samples (Frioux et al., 2020). Although this approach has some merits, several technological biases can result in partial taxonomic assignation, thus reducing the completeness of the analysis. For instance, 16S rRNA gene sequencing is often partial because it mostly targets a couple of hypervariable regions, although it is technically possible to target a nearly complete 16S rRNA sequence, depending on chosen primers and sequencing technology. Indeed, an extensive study by Johnson et al., revealed that a full 16S rRNA gene sequencing significantly improves the taxonomic resolution. Out of all of the partial sequencing tested, the V4 region performed worst, and the relative number of OTUs produced using the different sub-regions was not consistent depending on the identity threshold (Johnson et al., 2019). It has also been noted that the development of long-read sequencing platforms enhanced the accuracy of the sequencing thus improving the detection of single nucleotide polymorphisms (SNPs) in the complete 16S rRNA gene. Multiple copies of the 16S rRNA genes carrying unique SNPs can even be detected using this technique, allowing a strain-level identification (Johnson et al., 2019).

The thrive of metagenomic sequencing also benefited from the use of NGS to perform shotgun sequencing. A study by Ranjan et al. (2016) concluded that the use of shotgun sequencing compared to 16S sequencing significantly improved the diversity of bacterial species detected, thus allowing a finer prediction of the genes carried by a bacterial community. For instance, their study showed that more than 1,000 species of proteobacteria were only detected by shotgun sequencing performed on a stool sample and twice the amount of genes were predicted on average using one of the shotgun short-reads metagenomic technologies. The comparison of different short-read sequencing technologies, namely MiSeq and HiSeq, showed that the extra length produced by MiSeq sequencing (150–300 bp compared to 100 bp) enables a more specific identification of bacterial species and improves accuracy and completeness of MAGs that are essential to reliably predict the metabolic potential of the ecosystem (Ranjan et al., 2016; Frioux et al., 2020). A more recent study showed that the use of long-read technologies (e.g., PacBio) improved drastically the completeness of the MAGs and associated predicted genes: more than 98% of the genes predicted from PacBio-assembled MAGs were complete compared to only 40% using HiSeq-assembled MAGs (Xie et al., 2020). Indeed, the use of long reads enables the detection of repeated elements often found in ribosomal RNA genes or bacteriophage-related insertions that are frequently missed with short read sequencing (Derakhshani et al., 2020).

Once sequencing data have been acquired, the identification of species in the gut microbiome requires the assignment of the reads to taxonomies. At this step, not only is the quality of the reads important, but the accurate assignment to taxonomies also depends on the reference catalog used. For the assignment of 16S rRNA genes, two factors strongly influence results. First, there is high reliance on the diversity contained in the reference catalog and second, the amplified regions can skew the identification of OTUs. Indeed, 16S rRNA gene catalogs are often built from complete 16S sequences whereas the amplification and sequencing of the 16S rRNA gene usually targets hypervariable regions (V-regions), leading to biased performances and challenges when comparing different studies. To overcome this hurdle, a new tool called OTUX has been proposed, that combines several custom datasets of OTUs defined by 16S rRNA V-regions retrieved from full-length 16S rRNA sequences (Yadav et al., 2019). This new method was challenged against conventional full-length 16S databases and revealed an improved assignment of reads for all of the V-regions targeted, except for the V1–V3 region (Yadav et al., 2019). For the assignment of shotgun reads, using MAGs could significantly improve identification accuracy of bacterial species. Recent research efforts produced a comprehensive catalog containing more than 200,000 reference genomes from the human gut microbiome referenced as the UHGG database (Almeida et al., 2020). This work also revealed that 81% of the species in the catalog lacked a cultured representative, indicating the huge potential for discovering new keystone species in the future (Almeida et al., 2020).

Another critical parameter in the analysis of microbial communities using NGS is the sequencing depth. Several keystone species from the human gut microbiome, such as *C. minuta*, which presence in stool samples is significantly associated with BMI, are low abundance microbes that can be missed when using a reduced sequencing depth (Waters and Ley, 2019). Thus, an increased depth is required to ensure that the whole diversity of species from the gut microbiome is identified (Berry and Widder, 2014).

Considering data processing using bioinformatic tools, one of the main issues regarding the use of sequencing data to identify correlations between taxa within the gut microbiota is the compositional nature of these datasets. The analysis of read counts begins with their normalization by the total number of sequenced reads. As explained by Friedman and Alm, the estimation of correlation between parameters (e.g., species) is biased by the relationship between the fractions: because they must sum to 1, they are not independent (Friedman and Alm, 2012). Thus, a study can be artificially biased by the disappearance of highly abundant species due to technical or assignment issues: the relative abundance of low-abundant species will increase as the variables are dependent even if their absolute abundance is constant. Quantitative PCR (qPCR) is the predominant method to quantify biomass using 16S rRNA gene amplification, however, this method could significantly influence the models. Li et al. (2019) noticed a high variation between replicates when quantifying biomass from stool samples. Another flaw of this technique is due to the variable number of 16S rRNA gene copies in several microbial phyla such Firmicutes and Bacteroidetes, which results in an over-representation of such species. Friedman and Alm (2012) demonstrated that standard Pearson correlation estimation can falsely predict negative correlations between one dominant and several low abundant species because of the dependence between abundances. This issue has also been pointed by Berry and Widder (2014): they noted a loss of specificity in the co-occurrence networks when relative abundances were used. As a consequence, the SparCC method was developed to estimate the linear Pearson correlation between transformed variables: the log transformation of the ratio of abundances between a pair of OTUs because the ratio between abundances is independent from other OTUs included. Using SparCC, it was demonstrated that the bias observed in standard correlation studies that is induced by dominant species, is greatly reduced in simulated data of varying diversity. Applied to the HMP dataset, SparCC revealed new positive correlations between highly abundant and low abundant species, instead of the spurious negative correlations usually observed when using standard correlations (Friedman and Alm, 2012).

Recently, another method was developed by Li et al., to overcome the compositional bias when using generalized Lotka-Volterra (gLV) models. As explained above, gLV models are one of the most common approaches for modeling microbial interactions. These models also suffer from the compositional bias induced by the relative abundances. Indeed, absolute biomass values are needed in order to accurately fit the gLV differential equations model of each organisms’ growth rate to the data (Li et al., 2019). Therefore, Li et al. (2019) developed an algorithm called BEEM that estimates biomass *in silico* before inferring interactions when total biomass cannot be estimated experimentally. This algorithm introduces relative abundances in the equation modeling the growth rate of each species, resulting in two parameters that can be estimated using longitudinal datasets. This method was applied to diverse synthetic or existing datasets and accurately estimated biomass and gLVM parameters. It also allowed the identification of *F. prausnitzii* and *B. uniformis* as putative keystone species sharing numerous positive interactions with other bacteria (Li et al., 2019). Yet, this method is limited by available growth information for common species. Hence, only common dominant bacteria for which the information exists, may be accurately predicted.

Finally, recent developments have proposed to apply inferential statistics to network-based models in order to gain confidence in result interpretation. A study by Röttjers et al. (2021) recently proposed the use of null models to identify network properties that can be used to compare networks. They showed that among 20 networks built from time-series stool samples collected from 20 women, the new tool called anuran, could identify patterns that were found in 20–25% of the networks but only 3 associations were found in 10 networks or more, suggesting that associations between species or taxa may greatly vary from one individual to another (Röttjers et al., 2021). Although this is a proof-of-concept work applied to a limited population, this is an interesting approach to robustly identify keystone species based on stable interaction networks.

## Conclusion

In this review, we discussed the role of key gut microbes-derived metabolites in intestinal homeostasis, metabolic health, immune regulation, and gut-brain interactions. We next described current bioinformatic tools used in microbiome studies and highlighted weaknesses associated with some of these approaches for the identification of keystone species and their missing functions. We point for instance that commonly used bioinformatic tools that provide “presence or absence” information are not suitable in this regard as they fail to provide a view of species interactions. Network-based methods that calculate co-occurrence or co-abundance of species are thus more suited to predict keystoneness. However, these methods need to be complemented with approaches that reconstruct species-specific metabolic pathways, such as the Metage2Metabo algorithm, but in a system where nutrient, genomic information and metabolites output are simultaneously analyzed.

Our review has provided a snapshot of recent discoveries on gut microbiome metabolic activities and the current state of the field with respect to bioinformatics analysis of the microbiome. We propose that in the quest for keystone species, future studies should consider harmonization of sample and data processing and the integration of additional variables including age, gender, nutrition, and other environmental cues. It is also particularly important to provide mechanistic evidence supporting the functions of keystone species in modulating the microbiome ecosystem for instance through quorum sensing, cross-feeding, bacteriocins or through other as of yet unknown mechanisms. Such studies will set the stage to design microbiota-based therapeutic interventions to counter chronic diseases, by restoring keystone species and their beneficial effects on microbiome balance to support a healthy symbiotic interaction with their host.

## Author Contributions

All authors listed have made a substantial, direct and intellectual contribution to the work, and approved it for publication.

## Conflict of Interest

SC and HT are employees of YSOPIA Bioscience. The remaining author declares that the research was conducted in the absence of any commercial or financial relationships that could be construed as a potential conflict of interest.

## Publisher’s Note

All claims expressed in this article are solely those of the authors and do not necessarily represent those of their affiliated organizations, or those of the publisher, the editors and the reviewers. Any product that may be evaluated in this article, or claim that may be made by its manufacturer, is not guaranteed or endorsed by the publisher.
